# Pathogen Resistance Depending on Jacalin-Dirigent Chimeric Proteins Is Common among Poaceae but Absent in the Dicot Arabidopsis as Evidenced by Analysis of Homologous Single-Domain Proteins

**DOI:** 10.3390/plants12010067

**Published:** 2022-12-23

**Authors:** Lara Esch, Christian Kirsch, Lara Vogel, Jana Kelm, Nikolai Huwa, Maike Schmitz, Thomas Classen, Ulrich Schaffrath

**Affiliations:** 1 Department of Plant Physiology, RWTH Aachen University, 52056 Aachen, Germany; 2 Institute for Bioorganic Chemistry, Heinrich Heine University Düsseldorf, 52425 Jülich, Germany; 3 Institute for Bio- and Geosciences 1: Bioorganic Chemistry, Forschungszentrum Jülich, 52425 Jülich, Germany

**Keywords:** jacalin-related lectin, dirigent, chimeric protein, Rosetta stone theory, rice (*Oryza sativa*), Arabidopsis (*Arabidopsis thaliana*)

## Abstract

MonocotJRLs are Poaceae-specific two-domain proteins that consist of a jacalin-related lectin (JRL) and a dirigent (DIR) domain which participate in multiple developmental processes, including disease resistance. For *Os*JAC1, a monocotJRL from rice, it has been confirmed that constitutive expression in transgenic rice or barley plants facilitates broad-spectrum disease resistance. In this process, both domains of *Os*JAC1 act cooperatively, as evidenced from experiments with artificially separated JRL- or DIR-domain-containing proteins. Interestingly, these chimeric proteins did not evolve in dicotyledonous plants. Instead, proteins with a single JRL domain, multiple JRL domains or JRL domains fused to domains other than DIR domains are present. In this study, we wanted to test if the cooperative function of JRL and DIR proteins leading to pathogen resistance was conserved in the dicotyledonous plant *Arabidopsis thaliana.* In Arabidopsis, we identified 50 JRL and 24 DIR proteins, respectively, from which seven single-domain JRL and two single-domain DIR candidates were selected. A single-cell transient gene expression assay in barley revealed that specific combinations of the Arabidopsis JRL and DIR candidates reduced the penetration success of barley powdery mildew. Strikingly, one of these pairs, *At*JAX1 and *At*DIR19, is encoded by genes located next to each other on chromosome one. However, when using natural variation and analyzing Arabidopsis ecotypes that express full-length or truncated versions of *At*JAX1, the presence/absence of the full-length *At*JAX1 protein could not be correlated with resistance to the powdery mildew fungus *Golovinomyces orontii*. Furthermore, an analysis of the additional JRL and DIR candidates in a bi-fluorescence complementation assay in *Nicotiana benthamiana* revealed no direct interaction of these JRL/DIR pairs. Since transgenic Arabidopsis plants expressing *Os*JAC1-GFP also did not show increased resistance to *G. orontii*, it was concluded that the resistance mediated by the synergistic activities of DIR and JRL proteins is specific for members of the Poaceae, at least regarding the resistance against powdery mildew. Arabidopsis lacks the essential components of the DIR-JRL-dependent resistance pathway.

## 1. Introduction

Jacalin-related lectins (JRLs) are carbohydrate-binding proteins and they have been shown to be involved in regulatory processes and plant stress responses [1]. JRLs are conserved among various groups of organisms and were present before the divergence of monocots and dicots [2]. In the Brassicaceae and Poaceae families, respectively, a total number of 324 and 157 JRLs were identified [2]. In dicots, the majority of JRL proteins exist as mero- and hololectins, i.e., proteins consisting only of a single or multiple JRL domains, respectively, but JRLs are also part of chimeric proteins where they are combined with other domains [1]. In Arabidopsis, a total of 48 JRLs exist as mero-, holo-, or chimeric JRLs [3], while, in rice, Han et al. (2018) [2] identified 30 JRL proteins in total, from which 16 have a single JRL domain, 1 contains three repeated-JRL domains, and 13 JRLs occur in chimeric proteins. Chimeric JRL proteins are especially prevalent in the Poaceae family [2]. Chimeric proteins of JRL domains fused to dirigent (DIR) or nucleotide-binding adaptor shared by APAF-1, R proteins, and CED-4 (NB_ARC) domains exist exclusively in members of the Poaceae [1]. Rice, for example, has a total of seven proteins of these two types, with three of the NB_ARC- and four of the DIR-type [2]. Interestingly, all chimeric DIR-JRL proteins are located on rice chromosome 12, suggesting their origin was from gene duplication, possibly followed by neo-functionalization [4].

The function of the JRL domain has been studied extensively and shown to exhibit, for example, agglutination activity and binding to monosaccharides with varying specificity [5]. There have been various reports showing the involvement of JRL proteins in developmental processes and stress responses. The Arabidopsis protein *At*JAC1, i.e., a holo-jacalin with three JRL domains, positively controls flowering [6]. On the other hand, the two Arabidopsis mero-jacalins *At*JAX1 and *At*RTM1 contribute to early and late restriction of virus movement, respectively [7,8]. In rice, the single-domain JRL, SalT, has been shown to respond to abiotic stress [9]. Additionally, the constitutive expression of a wheat JRL, *Ta*JRLL1 consisting of two JRL domains, in transgenic Arabidopsis plants resulted in enhanced resistance against necrotrophic fungal pathogens, such as *Fusarium graminearum* and *Botrytis cinerea* [10]. Contrarily, the function of DIR-domain proteins is not well understood [5]. Initially, DIR proteins were thought to be involved solely in the stereospecific coupling of monolignols to yield lignans or modified lignin [11]. However, evidence for stress-induced expression of DIR proteins has suggested a role of these proteins in adaptive responses to various external stimuli [12].

Recently, a function in pathogen resistance was reported for chimeric DIR-JRL proteins from rice and wheat. Weidenbach et al. (2016) [4] have shown that *Os*JAC1, a chimeric rice DIR-JRL protein, is involved in broad-spectrum disease resistance against bacteria, oomycetes, and fungi when it is constitutively expressed in rice or barley. In addition, both *Os*JAC1 domains (JRL and DIR) were shown to recognize saccharide-containing mannose/glucose and galactose/galactobiose, respectively [13]. Wheat chimeric DIR-JRL proteins substantially diversified after separation from other cereal species and gained novel functionalities [14]. They are divided into three groups and *Ta*JA1 (syn. *Ta*MCJ1), *Ta*MCJ2, and *Ta*MCJ3 are members from these separate groups [5]. Overexpression of *Ta*JA1 (syn. *Ta*MCJ1) in transgenic tobacco significantly enhanced resistance against wildfire disease caused by the bacterium *Pseudomonas syringae*. This was not the case for the other two proteins *Ta*MCJ2 and *Ta*MCJ3 [5,15], indicating a divergence in function between the three groups. This is also reflected by the particular functions of the DIR-JRL wheat proteins *Ta*VER2, *Ta*WCI-1, and *Ta*Hfr-1 which play a role in vernalization, acquired resistance, and defense against insects, respectively (see Ma and Han 2021 and references therein).

In this study, we established that the cooperativeness of JRL and DIR domains in pathogen resistance is conserved in plants that originally contain chimeric monocotJRLs. The Rosetta stone theory proposes that the existence of such chimeric proteins predicts that proteins with the same domains but in separate proteins, as present in different species, function in the same biochemical pathway and may physically interact [16]. We challenged this hypothesis for Arabidopsis JRL and DIR proteins, and our results negate a cooperative function for JRL and DIR proteins in pathogen defense in Arabidopsis.

## 2. Results

### 2.1. Domain-Swap Experiments Reveal Functional Conservation for Domains of Chimeric monocotJRLs from Different Species

We have shown previously that constitutive expression of the rice monocotJRL *OsJAC1* in transgenic barley and wheat enhances resistance to powdery mildew. It was further demonstrated that both *Os*JAC1 domains, the N-terminal DIR and the C-terminal JRL domain, are required for pathogen resistance [4]. A phylogenetic analysis led to the identification of the *OsJAC1* orthologues *TaJA1* (syn. *TaMCJ1*, located on chr. 2B; see [5]) and *HvJAC1* (chr. H5, [4]) in wheat and barley, respectively. Transient overexpression (TOX) of *TaJA1* and *HvJAC1* in single barley epidermal cells, using particle bombardment, indicated that both proteins increased the resistance of transformed cells to barley powdery mildew (*Blumeria graminis* f.sp. *hordei*, *Bgh*), similar to *OsJAC1* [4].

**Figure 1:**
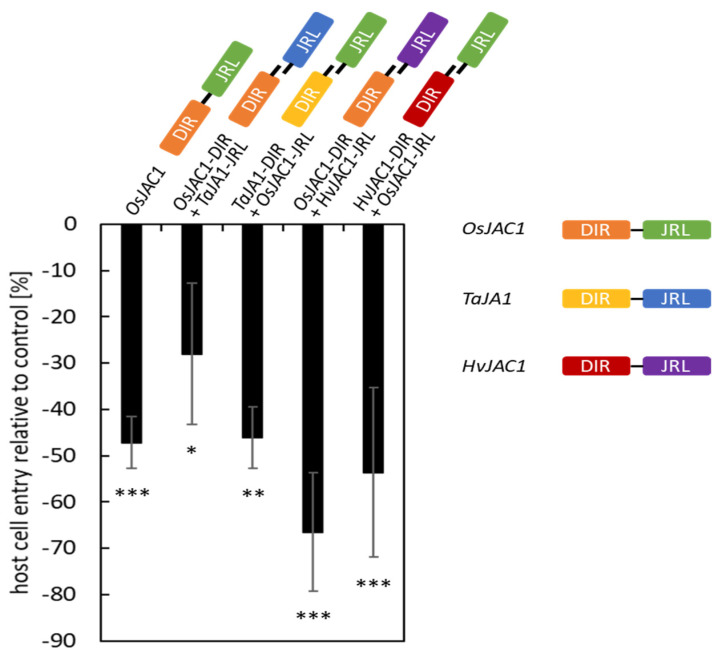
Domain-swap experiment with DIR and JRL domains of chimeric proteins from rice, barley, and wheat. Primary leaves of barley were transiently co-transformed with constructs encoding for the artificially separated domains of *Os*JAC1, *Ta*JA1, and *Hv*JAC1. At 48 h p.i., the effect of overexpression on the penetration success of *Bgh* was evaluated. Host cell entry rates were determined from the ratio of sites with successful penetration to the total number of interaction sites of spores with transformed cells. The negative control (empty vector) was set to 100%, and significant differences were determined using one-way ANOVA and are labeled with asterisks (*P* < 0.05: *; *P* < 0.01: **; *P* < 0.001: ***). The bars represent the mean values of at least three experiments with 50 to 300 evaluated interaction sites on three leaves (*n* = 3) ± standard deviation. For visualization, the values of the control are taken as baseline (equal to “0%”) and, hence, the negative values for the test candidates can be read intuitively as a reduction in penetration success.

To test if the cooperative function of the DIR and JRL domain of the monocotJRLs was conserved across species, we performed a domain-swap experiment, combining the DIR and JRL domains of *Os*JAC1 with the respective, heterologous domains of *Ta*JA1 or *Hv*JAC1. In a TOX assay, we confirmed that the transient overexpression of *OsJAC1* significantly lowered the rate of successful penetration of *Bgh* by 47%, compared to the control which was set to 0% (Figure 1). The penetration success of *Bgh* on barley epidermal cells transformed with an empty vector ranged from 27 to 45% in three independent experiments. The TOX of the constructs encoding for the separated *Os*JAC1-DIR or -JRL domain in combination with the respective domains of the barley orthologue *Hv*JAC1 resulted in a 67% and 54% reduction of *Bgh* penetration of transformed cells for the pairs of *Os*JAC1-DIR/*Hv*JAC1-JRL and *Hv*JAC1-DIR/*Os*JAC1-JRL, respectively. Similarly, the combination of *Ta*JA1-DIR/*Os*JAC1-JRL decreased the penetration efficiency to 46%. The transient overexpression of the *Os*JAC1-DIR domain and the JRL domain of *Ta*JA1 lowered the *Bgh* penetration rate less, but still significantly (−28%) (Figure 1). These results indicate that the function of the individual monocotJRL domains is highly conserved among the Poaceae species.

### 2.2. Transient Overexpression of Arabidopsis AtJAX1 and AtDIR19 Increases Resistance of Barley to Powdery Mildew, but AtJAX1 Is Not Required for Powdery Mildew Resistance in Arabidopsis

Chimeric proteins containing a JRL and DIR domain were reported to have evolved exclusively in monocotyledonous plants [1]. In Arabidopsis, by contrast, no such fusion proteins exist. Instead, we identified 50 proteins with JRL and 24 proteins with DIR domains (Figure S1). Out of them, a total of seven JRL and 22 DIR proteins contain only a single domain while the others have duplicated JRL domains or are fused to other functional domains. Weidenbach et al. (2016) [4] and the domain-swap experiment discussed above clearly demonstrated a cooperative effect in plant defense of single DIR or JRL domains of artificially separated chimeric monocotJRLs. Therefore, we tested if proteins from Arabidopsis with a single DIR or JRL domain also cooperate in a similar way.

**Figure 2:**
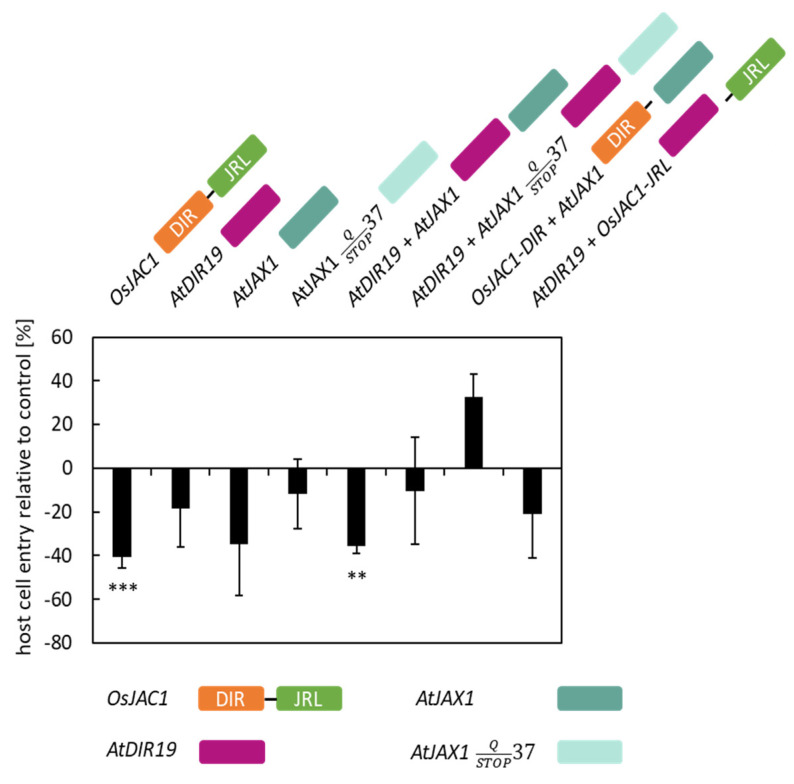
Testing of Arabidopsis candidate genes in a TOX assay. Primary leaves of barley were transiently transformed with constructs encoding for *At*DIR19 (*At1g58170*), *At*JAX1 *(At1g58160)*, and the variant *AtJAX1-STOP* and combinations thereof. The effect of transient expression on the penetration success of *Bgh* was evaluated at 48 h p.i. Host cell entry rates were determined from the ratio of sites with successful penetration to the total number of interaction sites of spores with transformed cells. The negative control (empty vector) was set to 100%, and significant differences were determined using the Mann–Whitney rank sum test and are indicated by asterisks (*P* < 0.01: **; *P* < 0.001: ***). The bars represent the mean values of at least three experiments with a total of 50 to 300 interaction sites evaluated on three leaves (*n* = 3) ± standard deviation. For visualization, the values of the control are taken as baseline (equal to “0%”) and, hence, the values of the test candidates can be read as differences in penetration success.

It has been established that neighboring genes are often co-regulated [17,18]. Interestingly, we identified that two genes encoding for a protein with a single DIR domain (*AtDIR19*, AtT1G58170) and a single JRL domain (*AtJAX1*, At1G58160), respectively, are located directly next to each other on chromosome 1 (Table 1). To evaluate a potential effect of these proteins in plant defense, we, again, used the TOX assay described above (Figure 2). The transient overexpression of the positive control (*OsJAC1*) significantly reduced cell penetration rate of *Bgh* by 40%, compared to the control which was set to 0% (penetration rate of *Bgh* in the control ranged between 22 and 48%). For *AtDIR19* and *AtJAX1*, overexpression did not significantly decrease the penetration rate of *Bgh*. The co-transformation of *AtDIR19* and the construct encoding for the JRL domain of *Os*JAC1 (*OsJAC1-JRL*), as well as *At*JAX1 and the construct encoding for separate DIR domain of *Os*JAC1 (*OsJAC1-DIR*), also did not lead to increased penetration resistance of transformed cells against *Bgh*. However, the co-expression of *AtDIR19* and *AtJAX1* significantly lowered the penetration rate of *Bgh* by 36%. From the literature data, it is known that the *AtJAX1* sequence of Col-0 contains a single-nucleotide polymorphism (SNP) leading to a premature stop at the amino acid position 37 [7] (Figure S2). The co-bombardment of this truncated version of *AtJAX1^STOP^* with *AtDIR19* did not significantly reduce the rate of penetration of *Bgh* on transformed cells (−10%) (Figure 2), indicating the full-length *At*JAX1 protein is necessary for *At*JAX1/*At*DIR19-mediated resistance of barley epidermal cells against the penetration of *Bgh*.

**Figure 3:**
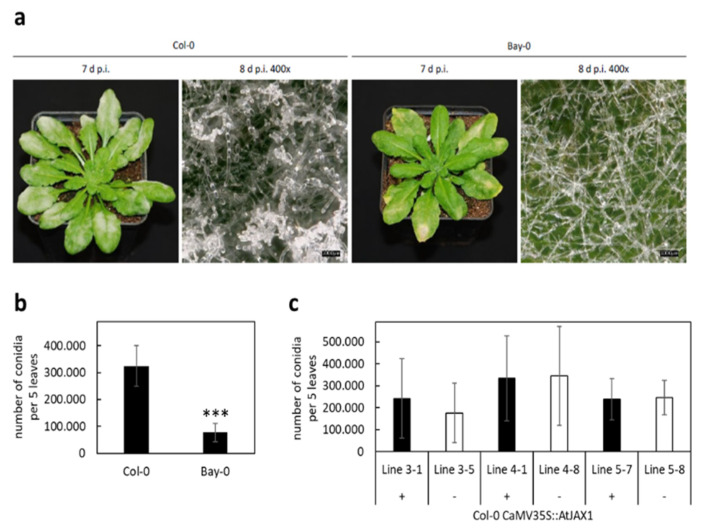
Disease severity of Arabidopsis Col-0 and Bay-0 inoculated with *Golovinomyces orontii*. Arabidopsis plants of ecotype Col-0 (*AtJAX1Stop*) and Bay-0 (*AtJAX1*) were inoculated with *G. orontii*. (**a**) Macroscopic evaluation was performed at seven and eight days post inoculation (d p.i.) as indicated, scale bars equal 100 µm. (**b**) Quantification of disease severity was performed by rinsing five infected leaves of twelve plants of each ecotype with water and counting of conidia at seven d p.i. (**c**) Disease severity of Col-0 plants complemented with a full length *AtJAX1* construct (+) and azygous sibling plants (−) was calculated from five infected leaves of each plant at seven d p.i. by rinsing with water and counting of conidia. Mean values are given in (**b**,**c**) ± standard deviation. Significant differences were determined using Student’s *t*-test and are indicated with an asterisk (*P* < 0.001: ***).

To test whether this pair of proteins also affected pathogen resistance in Arabidopsis, we used the database generated by the 1001 Genomes Project [19,20] and identified Bay-0 to have the full length *At*JAX1 protein, which was also reported by Yamaji et al. (2012) [7]. We inoculated Col-0 plants, expressing the truncated version of *At*JAX1, and Bay-0 plants with the Arabidopsis powdery mildew fungus *G. orontii* (Figure 3a). Interestingly, the leaves of Col-0 appeared more diseased than Bay-0 at seven days after inoculation. An observation at a higher magnification (400×) revealed a lower amount of conidia on the leaves of Bay-0 compared to the leaves of Col-0, and quantification of conidia from the infected leaves of both ecotypes confirmed this observation (Figure 3b).

To further explore the natural variation in *At*JAX1, we selected additional Arabidopsis ecotypes expressing the full-length *At*JAX1 protein (Cvi-0 and No-0) or the truncated *At*JAX1-STOP protein version (Kin-0 and La-0) (Figure S3). Quantification of conidia from the leaves infected with *G. orontii* at seven days post inoculation (d p.i.) showed that, while the pathogen was able to produce a significant higher number of spores on La-0, encoding for a truncated *At*JAX1-STOP protein, the number of conidia was reduced on Kin-0, also encoding for the truncated *At*JAX1-STOP protein. The number of conidia produced on Cvi-0 and No-0, expressing the full-length *At*JAX1, was similar to the number counted for Kin-0. This led to the conclusion that, at least for the tested ecotypes, the differences in resistance to *G. orontii* cannot be traced back to allelic variation of the *AtJAX1* gene. Interestingly, even though overexpression of *AtJAX1* and *AtDir19* in barley epidermal cells increased resistance to barley powdery mildew, the complementation of Col-0 with the full-length *AtJAX1* did not increase resistance to the Arabidopsis powdery mildew *G. orontii* as the number of conidia at 7 d p.i. was not significantly different between the transgenic and azygous control plants (Figure 3c). We therefore concluded that, in Arabidopsis, *At*JAX1 is not required for resistance to powdery mildew.

### 2.3. Several Pairs of Arabidopsis JRL and DIR Proteins Affect Resistance of Barley to Powdery Mildew

**Figure 4:**
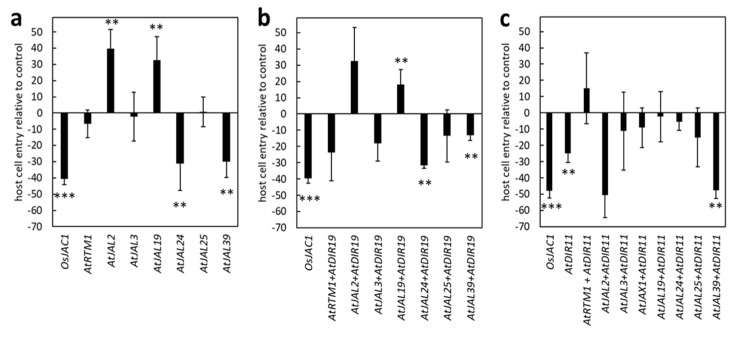
Transient expression of Arabidopsis DIR and JRL candidate genes. Primary leaves of barley were transiently transformed with the constructs as indicated. JRL-containing genes were expressed either alone (**a**), together with *AtDIR19* (**b**), or with *AtDIR11* (**c**). The effect of transient gene expression on the penetration success of *Bgh* was evaluated at 48 h p.i. Calculation of host cell entry rates and statistically analyses were performed in analogy to Figure 2. Significant differences (*P* = 0.01) were determined using the Mann–Whitney rank sum test and are indicated by asterisks (*P* < 0.01: **; *P* < 0.001: ***). The values presented are transformed to enable direct visualization of increased or reduced penetration success of *Bgh*. The bars represent the mean values of at least three experiments with a total of 50 to 300 interaction sites evaluated on three leaves (*n* = 3) ± standard deviation.

Using the TOX assay, we next tested the remaining six single-domain Arabidopsis JRL genes and one gene with three JRL domains for their ability to enhance resistance in barley against *Bgh*. The penetration rates of *Bgh* were reduced significantly when the plants were transformed with the constructs encoding for *At*JAL24 (−31%) and *At*JAL39 (−30%), compared to the control (set to 0%) (Figure 4a). This reduction was similar to the reduction observed in the plants transformed with *Os*JAC1 (−41%). Interestingly, the transient expression of the constructs encoding for *At*JAL2 (+40%) and *At*JAL19 (+33%) increased the cell entry rate, i.e., leading to reduced resistance. The transient overexpression of *At*RTM1, *At*JAL3, and *At*JAL25 did not influence *Bgh* penetration rates in this experiment.

Next, we examined whether there is a cooperative effect of Arabidopsis JRLs and the DIR protein *At*DIR19 on the resistance of barley epidermal cells to *Bgh*, as it was observed for *At*JAX1 and *At*DIR19. The TOX assay revealed that only the combinations of *At*DIR19/*At*JAL24 and *At*DIR19/*At*JAL39 resulted in a significantly reduced cell entry rate (−32% and −13%) compared to the control (Figure 4b). Co-transforming *AtDIR19* and *AtJAL19,* instead, increased the cell entry rate of *Bgh* by 18%. Interestingly, the co-expression of the selected JRL proteins with *At*DIR19 affected the *Bgh* penetration rate on transformed barley epidermal cells similar to what was observed for these single JRLs. This might indicate that *At*DIR19 only has a minor contribution to the observed *Bgh* resistance phenotype and negate the hypothesis of a cooperative effect between the proteins in question.

From the 22 single-domain DIR proteins (Figure S1), we selected *At*DIR11 (At1g22900) because it shares the highest similarity at the amino acid sequence level with the DIR domain of *Os*JAC1. *At*DIR11 was tested together with seven single-domain JRL candidates, including *At*JAX1, and with *At*JAL3 (hololectin with three JRL domains) in a complementary set-up as described above. Remarkably, *AtDIR11* significantly reduced the cell entry rate of *Bgh* (−25%) when expressed individually, although to a lesser extent than *Os*JAC1 (Figure 4c). This effect was enhanced by the co-expression of *AtDIR11* with the constructs encoding for *AtJAL2* and *AtJAL39,* which led to a reduction in cell entry rate of 50% and 48%, respectively. Particularly interesting was the reduction in cell entry rate associated with the co-expression of *AtDIR11/AtJAL2* (−50%) because this reversed the effect we observed for the single *AtJAL2* (+40%; Figure 4a) and the pair *At*DIR19/*At*JAL2 (Figure 4b).

### 2.4. Arabidopsis Protein Pairs AtDIR11/AtJAL2 and AtDIR11/AtJAL39 Do Not Physically Interact

**Figure 5:**
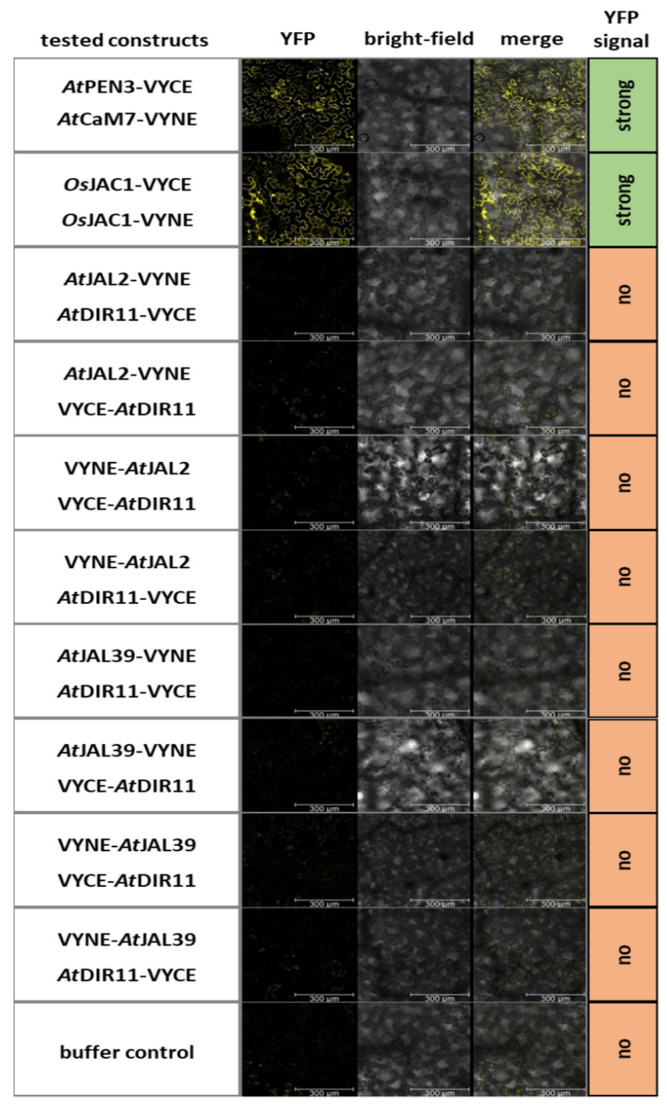
Bimolecular fluorescence complementation assay of Arabidopsis JRL and DIR candidates. Leaves of *N. benthamiana* were transformed via *Agrobacterium tumefaciens,* resulting in the co-expression of the constructs as indicated. The pairs *At*PEN3 and *At*CAM7, as well as *Os*JAC1/*Os*JAC1, served as the positive controls. An evaluation of YFP fluorescence (YFP) was performed using confocal laser scanning microscopy two days after the transformation at 20× magnification, excitation at 514 nm, and detection at 518–550 nm (*n* = 3). Additionally, bright-field pictures were taken, and merged images of the aforementioned pictures are shown for the localization of the YFP signal. Scale bar: 300 µm.

It was shown that separated DIR and JRL domains of *Os*JAC1 physically interact [4]. We therefore investigated whether the pairs *At*DIR11/*At*JAL2 and *At*DIR11/*At*JAL39, which both enhance powdery mildew resistance in barley, physically interact with each other in the dicotyledonous plant *Nicotiana benthamiana* in a bi-fluorescence complementation assay (BiFC) (Figure 5). Firstly, protein expression was confirmed using Western blot analysis (Figure S5). The protein pair *At*CaM7/*At*PEN3 [21] and *Os*JAC1, which was reported to form oligomeric species [13], were used as the positive controls. Strikingly, neither the combination of *At*DIR11/AtJAL2 nor *At*DIR11/*At*JAL39 resulted in YFP fluorescence, negating a physical interaction of these proteins (Figure 5). This contrasts the described interaction of artificially separated *Os*JAC1 domains [4]. The differences observed for the cooperativeness of JRLs and DIR proteins in dicots and monocots might indicate the involvement of other, yet unidentified, factors as part of the *Os*JAC1-resistance pathway that did evolve solely in monocotyledonous plants.

### 2.5. Constitutive Expression of OsJAC1 in Arabidopsis Does Not Affect Powdery Mildew Resistance

**Figure 6:**
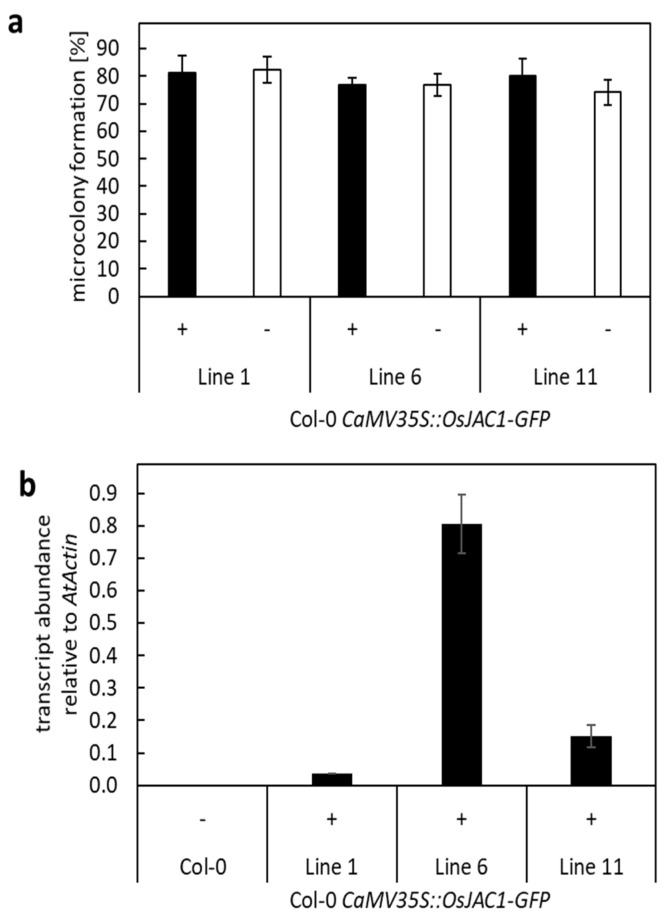
Transgenic Arabidopsis plants constitutively expressing *OsJAC1-GFP*. (**a**) Arabidopsis plants (Col-0) were transformed with *CaMV35S::OsJAC1-GFP* (Col-0 *CaMV35S::OsJAC1-GFP*) using *Agrobacterium tumefaciens*-mediated transformation. Four rosette leaves from three to four plants of different T1 lines (line1, line 6, and line 11) were inoculated with *G. orontii*. Transgenic plants (+) and azygous sibling plants (−) from the same events were used. Microcolony formation of *G. orontii* on these plants were examined microscopically after 48 h p.i. and calculated as the rate of successful invasion related to the total number of attacked cells. The mean values are shown in percent with standard deviation. No statistically significant differences were determined using Student’s *t*-test (*P* ≤ 0.96). (**b**) The transcript abundance of *OsJAC1-GFP* in plants of line 1, line 6, and line 11 was determined using qRT-PCR from the pooled RNA samples of four to five leaves and calculated relative to *At*Actin, according to Livak and Schmittgen (2001) [22]. The mean values from three technical replicates are shown with standard deviation.

So far, the TOX-experiments revealed that several pairs of DIR and JRL proteins are able to reduce cell entry rates of *Bgh* in barley epidermal cells. However, none of these pairs seem to physically interact with each other or mediate resistance in dicotyledonous plants. To further examine this observation, transgenic Arabidopsis plants that constitutively expressed a construct encoding for *Os*JAC1-GFP were generated. The functionality of the *Os*JAC1-GFP fusion protein in the resistance to powdery mildew had been demonstrated previously in a TOX-assay [4]. Plants from three independent lines were inoculated with *G. orontii*. Segregants, which did not contain the transgene, served as the control (azygous siblings). A microscopic quantification of micro-colony formation revealed no differences in the number of powdery mildew colonies between the *Os*JAC1-GFP transgenic and azygous plants (Figure 6a). Quantitative PCR confirmed *Os*JAC1-GFP expression in all transgenic lines, although at different expression levels (Figure 6b). The pronounced effect of *Os*JAC1-GFP on powdery mildew resistance in transgenic barley and wheat, and the lack of this effect in transgenic Arabidopsis plants, tempt us to speculate that a resistance pathway, similar to *Os*JAC1, does not exist in Arabidopsis, at least not against powdery mildew.

### 2.6. In Silico Analyses of Binding Properties of Selected JRL and DIR Domains

**Figure 7:**
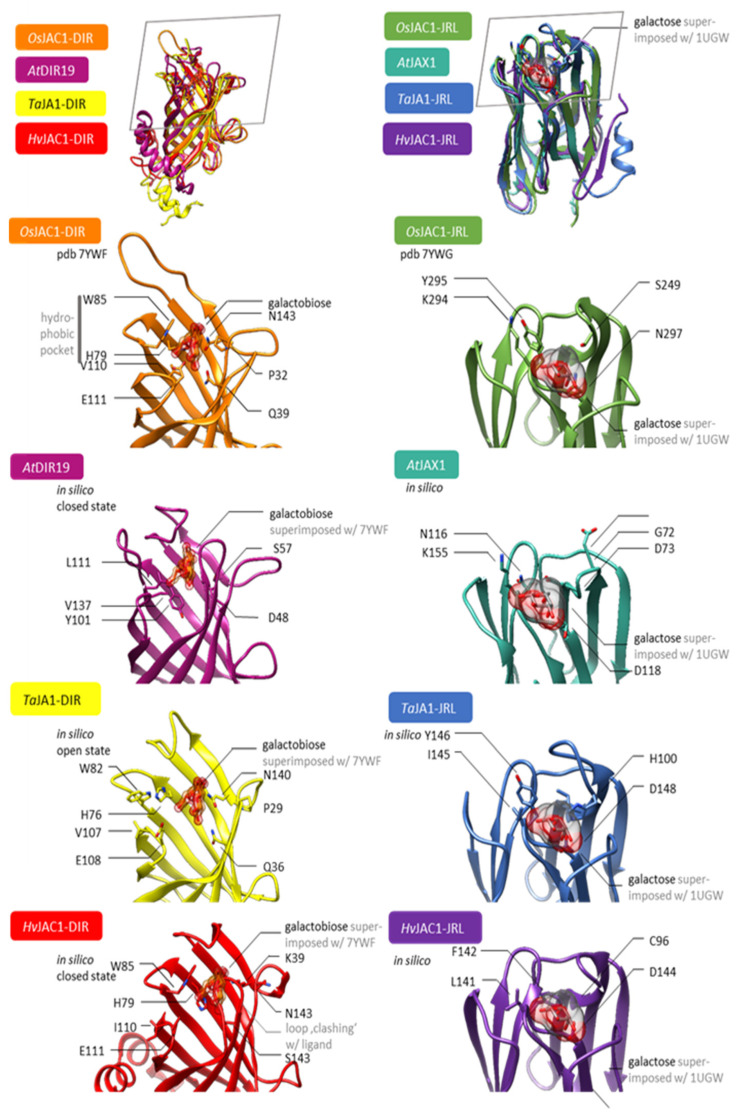
Structural models of selected DIR and JRL domains. Left column from top to bottom: Superposition of molecular models of *Os*JAC1-DIR [orange, from pdb 7YWF [23]], *Ta*JA1-DIR [yellow], *At*DIR19 [light purple], and *Hv*JAC1-DIR [red]. The ligand galactobiose is superimposed from OsJAC1-DIR to illustrate the putative binding site. Right column from top to bottom: Superposition of molecular models of *Os*JAC1-JRL [green, from pdb 7YWG], *Ta*JA1-JRL [blue], *At*JAX1 [turquoise], and *Hv*JAC1-JRL [purple]. The ligand is a superposition of galactose from pdb 1UGW [24]. The grey frame in the top row illustrates the selected perspective of the close-ups. The models for *Ta*JA1-DIR, *Hv*JAC1-DIR, *At*DIR19, *Ta*JA1-JRL, *At*JAX1, and *Hv*JAC1-JRL have been generated using TopModel.

Structural modelling was used to compare the DIR and JRL domains from different monocotJRLs to the single-domain DIR and JRL proteins of Arabidopsis. Although the native ligand of *Os*JAC1 is still unknown, crystallisation with surrogates has already shown that both domains, DIR and JRL, bind carbohydrates [23]. We hypothesized that similarities in the binding sites might point to a conserved mode of action for the aforementioned DIR and JRL domains. Molecular models were generated using the TopModel server. For the DIR domain, the sugar moiety (galactobiose) is bound by three feature sites: a bottom fixing the ligand via H-bond/polar interaction (Figure 7, *Os*JAC1-DIR Q39, N143), similarly a peripheral H-bond system (*Os*JAC1-DIR E111), and a hydrophobic pocket providing a π-system as the van der Waals interface, shielding the substrate from the solvent site (*Os*JAC1-DIR H79, W85, V110). Both DIR protein domains from *T. aestivum* and *H. vulgare*, i.e., *Ta*JA1-DIR and *Hv*JAC1-DIR, match these three features sufficiently, so that the putative molecular interactions could be satisfied. In the case of *At*DIR19, the binding site appears altered to some extent, compared with those of its Poaceaen pendants. The hydrophobic pocket in *At*DIR19 is formed by Y101, L111, and V137. Immanently, the shielding tryptophan is missing. The bottom H-bond system is given by D48. Together with the fact that there is a cavity for a putative carbohydrate ligand, the aberrant, but similar, binding site might render the very same mode of action as in the Poaceaen homologs. All structures have been modelled as apo-form. The ligand shown in Figure 7 is only a superimposition from pdb 7YWF [23] to highlight the binding site. However, the structures from *Ta*JA1-DIR and *Hv*JAC1-DIR could well represent the mechanistic intermediates. For example, the binding site of *Ta*JA1-DIR is more open compared to the holo-binding site of *Os*JAC1-DIR. This can be seen particularly well in W82, which could not fulfil its function as a solvent shield here. This could be an open conformation that favours ligand binding. In contrast, the binding site in *Hv*JAC1-DIR is closed to the extent that the sugar cannot bind at all. It could be speculated that these are the intermediates of a conformational selection mechanism with which even a large carbohydrate ligand can be specifically bound. The DIR domain of *Os*JAC1 exhibits a previously unknown carbohydrate binding property [13,23]. The similarity in the homologues underline that these are true orthologues, both structurally and functionally.

The carbohydrate binding for the lectin-related domain depends on two binding features: Polar interactions to the hydroxy groups are satisfied via the amid-backbone of a complementarily shaped loop. Furthermore, van der Waals interactions are provided by S249, Y295, and K294. In *Os*JAC1, there is only a bottom polar interaction provided by N297. To put it in a nutshell, *Ta*JA1 and *Hv*JAC1 provide these features as well as including a similarly shaped loop, forming the binding-site ground. Seemingly, there is no induced fit or conformational selection upon binding. In contrast, *At*JAX1 comprises a different binding site. On the one hand, the loop, which is homologous to K294/Y295 in *Os*JAC1-JRL, namely K115/N116, is softly bent towards the putative saccharide-binding site. This might be seen as a closed state because, after all, amino acids with similar physicochemical properties as in the homologues are found at this position. On the other hand, the loop, which is homologous to S249 in *Os*JAC1, namely D71-D73, is significantly shorter in *At*JAX1 compared to their homologues. All together, these features might point to mechanistic differences mediated by the JRL domains of AtJAX1 and those of the monocotsJRLs.

## 3. Discussion

Flowering plants can be grouped into monocots and dicots which are estimated to have split from a common ancestor approximately 200 million years ago. Today, and as result of the separation, species of both groups differ in habitus and organ morphology and are not interfertile. It is worthy to notice that, despite the monocot–dicot split dating back a long time, some components of the plant immune response are conserved in both groups of plants. This is true, e.g., for the barley *Hv*MLO (mildew locus o) and Arabidopsis *At*MLO2 proteins which both function as susceptibility factors in powdery mildew diseases [25,26]. Signaling components acting downstream of the pattern recognition receptors *Os*Xa21 (*Xanthomonas oryzae* pv. *oryzae* resistance 21) and *At*ERF (ethylene response factor) have also been shown to be conserved, indicating their importance in essential functions [27,28]. On the other hand, the immune receptors known as TIR-NBS-LRRs (toll/interleukin-1 receptor-nucleotide binding site-leucine-rich repeat, TNLs), for example, have evolved, and expanded massively, in dicots only, while being absent in monocots [29].

*Os*JAC1 is a two-domain protein consisting of a DIR and a JRL domain and is involved in broad-spectrum disease resistance of rice [4]. This class of chimeric proteins exists exclusively in monocots [1]. In dicots, by contrast, these respective domains are only present in separate proteins. This observation raised the question whether particular members of those single-domain proteins of dicots function cooperatively as it was shown for artificially separated domains of *Os*JAC1. We addressed this question in two subsequent blocks of experiments by firstly verifying whether artificially separated domains of *Os*JAC1 interact with the respective domains of heterologous proteins from wheat or barley, and by secondly testing the combinations of selected Arabidopsis single-domain JRLs and DIR proteins for a mutual contribution to plant resistance.

It has been shown previously that the constitutive expression of *OsJAC1* not only enhances disease resistance in rice but also in wheat and barley, which indicates that all components necessary for *Os*JAC1-mediated resistance are shared between those monocots [4]. It has been demonstrated further that the co-expression of constructs encoding for separated JRL or DIR domains of *Os*JAC1 in a TOX assay is sufficient to enhance powdery mildew resistance in barley [4]. In a domain-swap experiment with constructs encoding for single domains of *Os*JAC1, and its potential orthologs *Ta*JA1 and *Hv*JAC1, all domain pairs tested lowered the success rate of penetration of *Bgh*, although to different extents (Figure 1). Interestingly, the heterologous combinations of JRL and DIR domains from rice and barley increased the penetration resistance of barley to powdery mildew more than the combination of single-domain proteins from rice and wheat or the full-length, wild-type *Os*JAC1.

We selected Arabidopsis genes encoding JRL or DIR proteins based on their domain composition and sequence similarity to *Os*JAC1, and by literature mining (Table 1). Strikingly, one pair, *AtJAX1* and *AtDIR19,* is located directly next to each other on chromosome 1. It has been suggested that neighboring genes can be involved in common biological processes, such as metabolic clusters [30]. In addition, a function for *At*JAX1 in defense against potexviruses was already published [7]. Using the TOX assay in barley [31], we found that only the co-expression of *AtJAX1* and *AtDIR19*, but not the expression of single genes, lowered the penetration rate of *Bgh* to a similar extent as that observed for the *OsJAC1* gene (Figure 2). To further substantiate this observation, we made use of a naturally occurring allelic variation of the *AtJAX1* gene [7]. In Col-0, this allelic variation leads to a premature STOP. The co-expression of this shorter version of the protein together with *AtDIR19* abolished the effect on the penetration rates of *Bgh* (Figure 2). These results suggested a cooperative effect of the full-length *At*JAX1 and *At*DIR19 in the resistance of barley to *Bgh*, similar to what was previously shown for the artificially separated domains of *Os*JAC1 [4]. To see if *At*JAX1 and *At*DIR19 are also involved in the resistance of the dicot Arabidopsis against powdery mildew, we inoculated Bay-0 and Col-0 plants, containing a full-length and a truncated version of *At*JAX1, respectively, with *G. orontii.* Both Arabidopsis ecotypes showed mildew disease symptoms, however, the number of conidia was reduced on Bay-0 (Figure 3). This result seemed to point to a role for *At*JAX1 in powdery mildew resistance of barley and Arabidopsis. Surprisingly, the transgenic Col-0 lines complemented with the full-length *At*JAX1 did not differ in their resistance to powdery mildew compared to the azygous control lines (Figure 3). An investigation of additional Arabidopsis ecotypes, which also either express the truncated or full-length *At*JAX1, also indicated that there was no correlation between the presence of the full-length *At*JAX1 and resistance to powdery mildew in Arabidopsis (Figure S3). Because previous studies suggested that *At*JAX1 and the JRL *At*RTM1 contribute to early and late restriction of virus movement, respectively [7,8], we concluded that there are mechanistic differences in JRL-associated resistance against different classes of pathogens in Arabidopsis. Similarly, the results from the structural modelling account for the similarities, but also the differences, among the DIR and JRL domains from the monocotJRLs in comparison to *At*DIR19 and *At*JAX1 (Figure 7). This is especially true for the hydrophobic pocket of *At*DIR19, formed by Y101, L111, and V137, which appears different to those of monocotJRLs because the shielding tryptophan is missing. In the case of *At*JAX1, structural differences make the binding of a different ligand likely.

Although *At*JAX1 was able to increase resistance to *Bgh* when transiently overexpressed in barley in combination with *At*DIR19, in Arabidopsis, *At*JAX1 did not seem to be involved in the resistance to powdery mildew. We, therefore, tested more single-domain Arabidopsis JRL and DIR proteins in combination and identified additional pairs that were able to enhance resistance to barley powdery mildew when co-expressed in barley (Figure 4). In the dicot plant *N. benthamiana*, however, a BiFC experiment revealed that these pairs did not physically interact with each other (Figure 5). This result led to the conclusion that, while JRL and DIR proteins in a monocotyledonous plant, such as barley, are able to function cooperatively to increase disease resistance to powdery mildew, this function does not seem to be conserved in dicot plants, such as *N. benthamiana* and Arabidopsis. This was confirmed when transgenic Arabidopsis plants constitutively expressing *OsJAC1-GFP* did not show enhanced resistance against powdery mildew (Figure 6). Because the overexpression of *Os*JAC1 in Arabidopsis was reported to increase resistance against ionizing radiation [32], a general incapability of *Os*JAC1 functionality in Arabidopsis can be excluded. Rather, it seems that, in dicots, resistance to powdery mildew might be independent of *Os*JAC1 or, more generally, *Os*JAC1-like JRL and DIR proteins. Therefore, we concluded that monocotyledonous plants, such as rice, barley, and wheat, share downstream components required for *Os*JAC1-mediated resistance which are lacking in the dicot Arabidopsis. This, in turn, might also be the reason why BiFC in *N. benthamiana* did not work for the candidate protein pairs tested. *Ta*JA1 (syn. *Ta*MCJ1), the wheat ortholog of *Os*JAC1, enhances resistance against wildfire disease caused by the bacterium *P. syringae* in transgenic tobacco [5]. Therefore, the functionality of Poaceae-specific DIR–JRL chimera in pathogen defense of dicots cannot be negated in general. More likely, it seems that the DIR–JRL resistance pathway is specific for particular combinations of plant species and certain diseases.

## 4. Materials and Methods

### 4.1. Plant Material, Fungal Isolates, and Inoculation

Barley cv. Ingrid (MPI for Plant Breeding Research, Cologne) was grown in soil in controlled environmental conditions (18 °C, 65% relative humidity, 16 h photoperiod, and 200–250 μmol photons s^−1^ m^−2^ light intensity). *Blumeria graminis* f. sp. *hordei* (*Bgh*, Isolat K1) [33] was received from Paul Schulze-Lefert (MPI for Plant Breeding Research, Cologne) and cultivated on barley cv. Ingrid*Mlo* at 18 °C, 65% relative humidity, a 16-h photoperiod, and 130–150 μmol photons s^−1^ m^−2^ light intensity. The inoculation was performed as described in Weidenbach et al. (2014) [34]. *Bgh* infection success was quantified 48 h after inoculation using a light microscope. Leaf chlorophyll was removed with ethanol, chloroform (4/1; *v*/*v*), and 0.15% trichloroacetic acid. Fungal structures were stained with ink acetic acid (10% blue ink, 25% acetic acid). A minimum of 50–100 infection sites were counted per leaf and categorized.

For pathogen assays, *Arabidopsis thaliana* ecotypes Col-0, Bay-0, Cvi-0, No-0, Kin-0, and La-0 (collection RWTH Aachen University) and their respective overexpression lines were grown in soil in a controlled environment (22 °C, 60% relative humidity, 8.5 h photoperiod, and 120 μmol photons s^−1^ m^−2^ light intensity). *Golovinomyces orontii* (Ralph Panstruga, RWTH Aachen University) was cultivated on Arabidopsis plants Col-0 or *eds1-2* at 20–22 °C, 50–60% relative humidity, a 16-h photoperiod, and 80 μmol photons s^−1^ m^−2^ light intensity. The inoculation was performed via leaf-to-leaf inoculation or by brushing conidiospores from infected leaves through a 100 µm sieve onto detached leaves, which were placed on water agar (1% [*w*/*v*] agar-agar) in a 120 cm (height) settling tower at an average spore density of five conidiospores mm^−2^. After the settling of conidiospores for 1 h, the plants or detached leaves were returned to the growth cabinets and cultivated under the conditions described above. *G. orontii* infection was quantified seven days after inoculation by washing off conidiospores of five infected leaves. The leaves were vortexed for 1 min with 30 mL 0.1% Tween-20 solution in a 50 mL tube. The leaves were then discarded, and the spore suspension was centrifuged at 4000× *g* for 20 min. The supernatant was discarded by slowly aspirating from the top and spores were resuspended in 500 µL of the remaining supernatant. Spores were counted using a counting chamber (Thoma Haemocytometer).

The overexpression lines *CaMV35S::AtJAX1* and *CaMV35S::OsJAC1-GFP* in the Col-0 background were stably transformed using the floral dip method with *Agrobacterium tumefaciens* cultures, as described in Zhang et al. (2006) [35], carrying the constructs AtJAX1-pEarleyGate100 and OsJAC1-pEarleyGate103, respectively. *AtJAX1* was amplified from Arabidopsis genomic DNA flanked with attB1 and attB2 recombination sites and *OsJAC1* was previously cloned [4]. The fragments were cloned into pEarleyGate100 and pEarleyGate103, respectively [36], using the Gateway technology (Invitrogen). For reproduction, the Arabidopsis plants were transferred to controlled conditions at 22 °C, 60% relative humidity, a 16-h photoperiod, and 110 μmol photons s^−1^ m^−2^ light intensity. Abundance of transcripts from the transgene were quantified by qRT-PCR using a 7300 Real Time PCR System (Applied Biosystems, Foster City, California, USA) and the Platinum SYBR Green qPCR SuperMix-UDG with ROX (Invitrogen, Carlsbad, USA). The parameters were set according to manufacturer’s data (50 °C for 2 min, 95 °C for 10 min, 95 °C for 15 s, and 60 °C for 1 min, using 40 cycles and followed by melting point analysis).

### 4.2. Transient Overexpression Assay (TOX) on Single Barley Cells

The TOX assay was performed as described in Weidenbach et al. (2016) [4] and Schweizer et al. (1999) [31]. In short, the coding sequences (CDS) of the respective genes were obtained by PCR using cDNA of the corresponding plant and primers, as indicated in the supplement. Using restriction digestion enzymes, CDS was cloned into pIPKTA09 behind the CaMV 35S promoter. The primary leaves of seven-day-old barley plants (Ingrid*MLO*) were co-transformed via gold particle bombardment with the pIPKTA09 vector carrying the gene(s) of interest and a second vector (pUBI:GUS), leading to the expression of the β-glucuronidase (GUS) reporter gene which enabled the identification of transformed cells. At 24 h after bombardment, the leaves were inoculated with *Bgh* and GUS-stained after an additional 48 h. The surface structures of *Bgh* were stained with ink-acetic acid solution (10% blue ink, 25% acetic acid) and analyzed using light microscopy. Therefore, cells which successfully blocked *Bgh* penetration and cells which were invaded by the pathogen were categorized and the data were used to calculate the cell entry rates of *Bgh*. Firstly, statistical analysis was performed with these data. Thereafter, for the sake of simplicity, the results are shown in plots in which the control was set to 0%. In this way, the effect of a test candidate gene to reduce the penetration success of *Bgh* could intuitively be obtained from the plotted data as negative value. A list of primers is available in Table S1.

### 4.3. Identification of JRL- or DIR-Domain-Containing Proteins in Arabidopsis

Jacalin-related lectin (JRL) and DIR protein candidates from *Arabidopsis thaliana* were identified using the Protein Search function (PF01419 and PF03018 as query) and the BLAST-Tool of The Arabidopsis Information Resources (TAIR, [37]. Additionally, the NCBI Batch Web CD search tools was used [38,39]. The numbers obtained in our study slightly differ from those published earlier by other groups [3,12].

The phylogenetic tree of Arabidopsis JRL and DIR proteins was constructed in MEGA7 based on ClustalW alignment using a Maximum Likelihood method based on the Jones–Taylor–Thornton Matrix model with 1000 replicates to retain bootstrap values [40,41,42,43].

### 4.4. Bi-Fluorescence Complementation Assay (BiFC)

*Nicotiana benthamiana* plants (Thomas Lahaye, Eberhard Karls University, Tübingen) were grown in soil in a controlled environment (24 °C, 75% relative humidity, 15-h photoperiod, and 350 μmol photons s^−1^ m^−2^ light intensity). The leaves of 4–6-week-old *N. benthamiana* plants were co-infiltrated using a syringe with *A. tumefaciens* strain *AGL1* carrying the pDEST-^GW^VYNE, pDEST-VYNE(R)^GW^, pDEST-^GW^VYCE, or pDEST-VYCE(R)^GW^ vector with the corresponding gene [44]. Infiltrated leaf area was cut out two days after treatment and vacuum infiltrated with water. The evaluation of BiFC signal (restored YFP) was performed using a Leica SP8 confocal laser scanning microscope (objective: HC PL APO CS2 20×/0.75 IMM, excitation at 514 nm using an Argon laser, and detection at 518–550 nm 514 nm)

### 4.5. Phenolic Total Protein Extraction and Western Blot

Protein extraction was performed as described in Thomas et al. (2015) [45] using approximately 150 µL of ground leaf tissue of *N. benthamiana.* The protein samples were mixed with a 2× protein gel loading buffer (NuPAGE^TM^ LDS Sample Buffer (4X) and NuPAGE^TM^ Sample Reducing Agent (10X)) and incubated at 95 °C for 10 min. Next, the samples were separated on a Bis–Tris gel as described in Huwa et al. (2021) [13], and the proteins were transferred onto nitrocellulose membranes for immunodetection using Anti-HA (rabbit, 1:1000, Cell Signaling Technology, Danvers, MA, USA) and Anti-cMyc antibodies (mouse, 1:2000, obtained from U. Commandeur, RWTH Aachen) as the primary antibodies. Anti-rabbit-HRP (goat, 1:2000, Cell Signaling Technology, Danvers, MA, USA) and Anti-mouse-HRP (horse, 1:2000, Cell Signaling Technology, Danvers, MA, USA) were used as the secondary antibodies.

### 4.6. Structural Modelling

Molecular models for *Ta*JA1-DIR, *Hv*JAC1-DIR, *At*DIR19, *Ta*JA1-JRL, *At*JAX1, and *Hv*JAC1-JRL were generated using the TopModel server, a template-based, deep-learning algorithm [46,47]. The molecular models of *Os*JAC1-DIR (pdb 7YWF) and *Os*JAC1-JRL are obtained from Huwa et al. (2022) [23]. The ligand for the JRL proteins is a superposition of galactose from pdb 1UGW [24].

## Figures and Tables

**Table 1:** Arabidopsis DIR and JRL candidate genes tested in this study.

Dirigent	JRL (# Domains)	Protein ID	Domain	*Os*JAC1 Sequence Similarity (*E*-Value)
	At1G05760 (1)	RTM1	Jacalin-like superfamily	-
	At1G05770 (1)	JAL2	Jacalin-like superfamily	-
	At1G19715 (3)	JAL3	Jacalin-like superfamily	3 × 10^−12^
			Jacalin-like superfamily	
			Jacalin-like superfamily	
At1G22900		DIR11	Dirigent superfamily	0.00006
	At1G58160 (1)	JAX1	Jacalin-like superfamily	7.1
At1G58170		DIR19	Dirigent superfamily	-
	At1G73040 (1)	JAL19	Jacalin-like superfamily	5 × 10^−14^
	At2G43730 (1)	JAL24	Jacalin-like superfamily	0.48
	At2G43740 (1)	JAL25	Jacalin-like superfamily	-
	At3G59620 (1)	JAL39	Jacalin-like superfamily	-

Arabidopsis DIR and JRL domains were identified via a NCBI Batch Web CD search. To determine sequence similarity, a TAIR protein BLAST was performed using *Os*JAC1 as the query. The table shows the E-values (smallest number of hits obtained at random in the search for similar sequences to *Os*JAC1 in the TAIR database). Gene identification numbers (Gene ID) of respective Arabidopsis DIR and JRL candidate genes are given. Protein names are given as well and the numbers in brackets indicate the number of JLR domains per protein.

## Data Availability

The data supporting the findings of this study are available from the corresponding author upon reasonable request.

## Supplementary Materials

The following supporting information can be downloaded at https://www.mdpi.com/article/10.3390/plants12010067/s1, Figure S1: Localization of the JRL and DIR genes across Arabidopsis chromosomes; Figure S2: Sequence analysis of *AtJAX1* from Col-0 and Bay-0; Figure S3: Disease severity of Arabidopsis ecotypes La-0, No-0, Cvi-0, and Kin-0 inoculated with *G. orontii*; Figure S4: Molecular phylogenetic analysis of DIR and JRL proteins of *Arabidopsis thaliana* and MonocotJRLs using a Maximum Likelihood method; Figure S5: Protein abundance in the BiFC experiment with AtDIR11 and AtJAL2/AtJAL39; Table S1: Primer used in this study.
