# Supplementary material for: Pathogen Resistance Depending on Jacalin-Dirigent Chimeric Proteins Is Common among Poaceae but Absent in the Dicot Arabidopsis as Evidenced by Analysis of Homologous Single-Domain Proteins
Source: Plants (Basel). 2022 Dec 23;12(1):67. doi: 10.3390/plants12010067 (PMC9824508; doi:10.3390/plants12010067)
Supplement: Supplementary file 1 [file plants-12-00067-s001.zip › plants-2079538-supplementary.pdf]

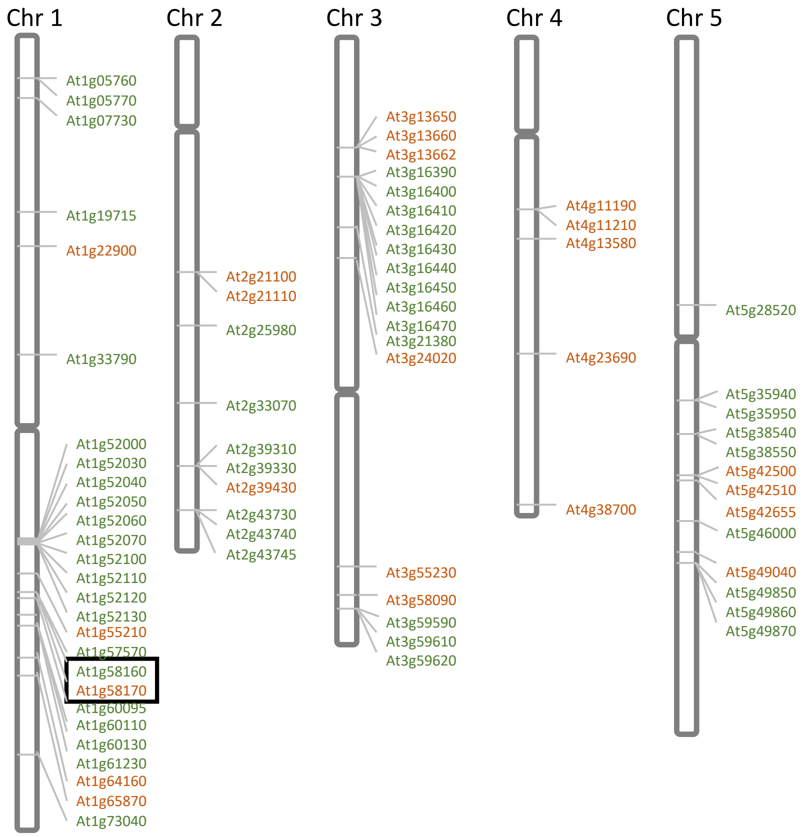

Figure S1: Localization of the JRL and DIR genes across Arabidopsis chromosomes

The scheme shows the identification numbers of all genes encoding of proteins with JRL (PF014199) or DIR domain (PF03018) in green or orange, respectively. The figure was generated using the 'Chromosome Map Tool' of TAIR (https://www.arabidopsis.org). Boxed is a pair of JRL and DIR genes located next to each other on chromosome 1.

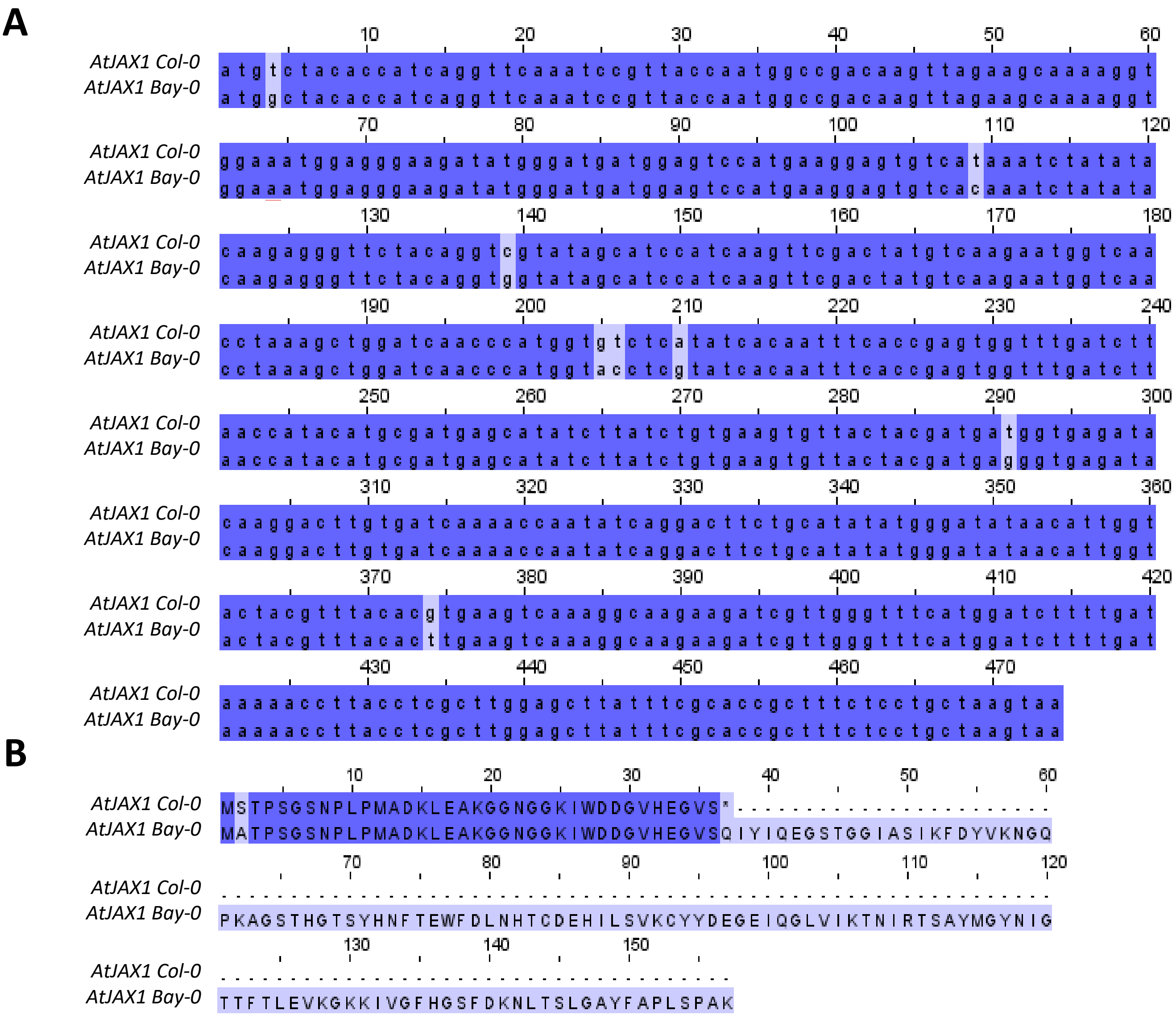

Figure S2: Sequence analysis of *AtJAX1* from Col-0 and Bay-0

Alignments of *AtJAX1* cDNAs from Col-0 and Bay-0 are shown in A). The corresponding amino acid sequences are given in B). For Col-0 a translational termination codon was found at position 109-111 (A) leading to a truncated version of the *At*JAX1 protein B). Figure was taken from Yamaji et al. (2012) and re-constructed.

Yasuyuki Yamaji, Kensaku Maejima, Ken Komatsu, Takuya Shiraishi, Yukari Okano, Misako Himeno, Kyoko Sugawara, Yutaro Neriya, Nami Minato, Chihiro Miura, Masayoshi Hashimoto, Shigetou Namba, Lectin-mediated resistance impairs plant virus infection at the cellular level, The Plant Cell, Volume 24 (2) 2012, Pages 778–793, https://doi.org/10.1105/tpc.111.093658

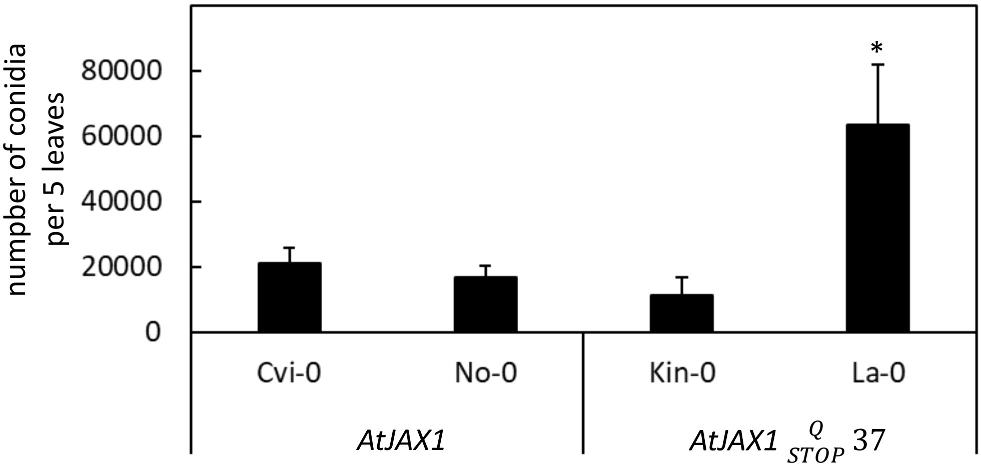

Figure S3: Disease severity of Arabidopsis ecotypes La-0, No-0, Cvi-0 and Kin-0 inoculated with *G. orontii*

Quantification of disease severity was done by rinsing five infected leaves of different ecotypes with water and counting of conidia at seven days after inoculation. Mean values are given ± standard deviation. Significant differences were determined by One Way Anova and indicated with an asterisk ($P<0.05$: *).

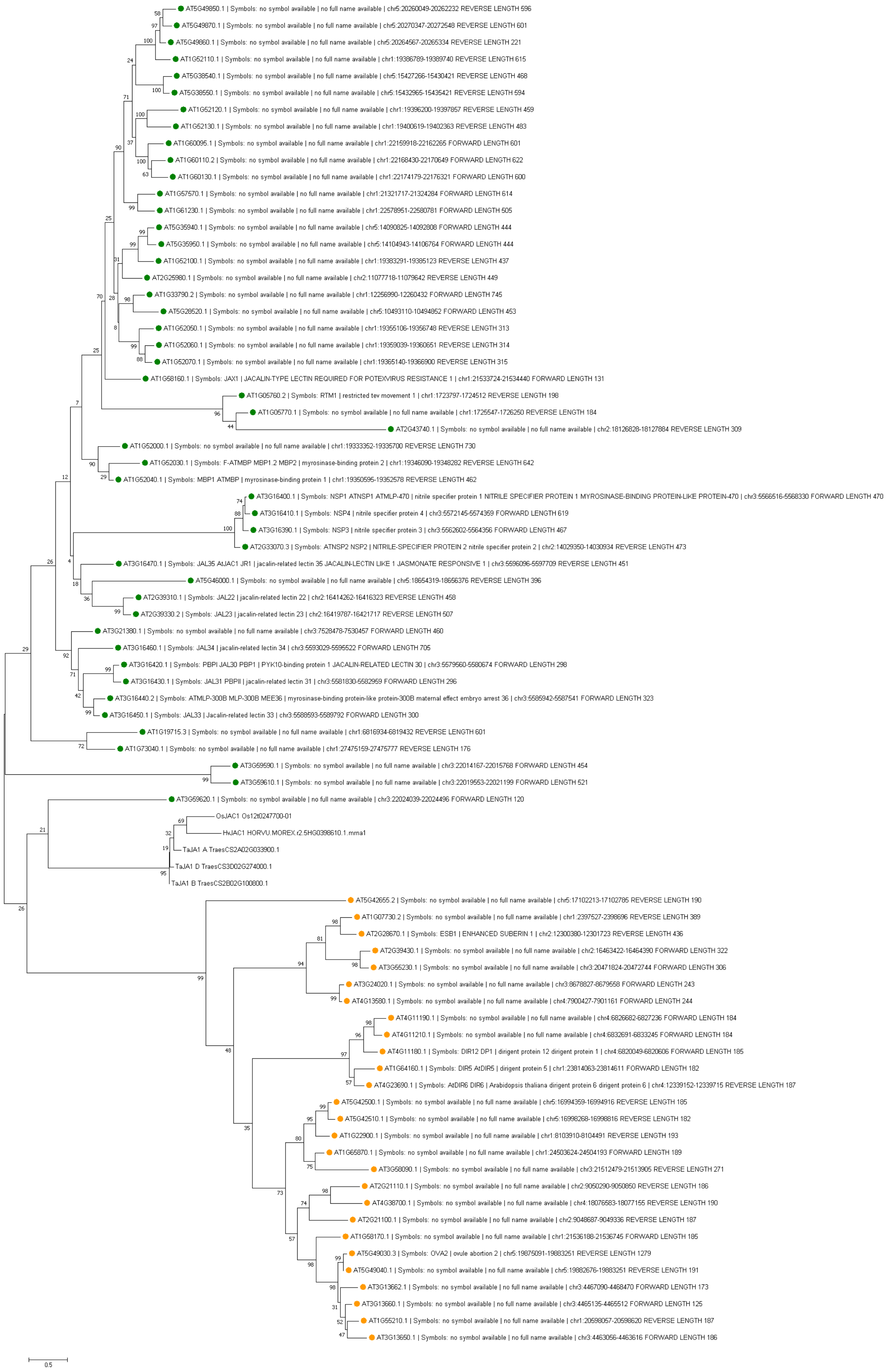
AT5G49850.1 | Symbols: no symbol available | no full name available | chr5:20260049-20262232 REVERSE LENGTH 596
AT5G49870.1 | Symbols: no symbol available | no full name available | chr5:20270347-20272548 REVERSE LENGTH 601
AT5G49860.1 | Symbols: no symbol available | no full name available | chr5:20264567-20265334 REVERSE LENGTH 221
AT1G52110.1 | Symbols: no symbol available | no full name available | chr1:19386789-19389740 REVERSE LENGTH 615
AT5G38540.1 | Symbols: no symbol available | no full name available | chr5:15427266-15430421 REVERSE LENGTH 468
AT5G38550.1 | Symbols: no symbol available | no full name available | chr5:15432965-15435421 REVERSE LENGTH 594
AT1G52120.1 | Symbols: no symbol available | no full name available | chr1:19396200-19397857 REVERSE LENGTH 459
AT1G52130.1 | Symbols: no symbol available | no full name available | chr1:19400619-19402363 REVERSE LENGTH 483
AT1G60095.1 | Symbols: no symbol available | no full name available | chr1:22159918-22162265 FORWARD LENGTH 601
AT1G60110.2 | Symbols: no symbol available | no full name available | chr1:22168430-22170649 FORWARD LENGTH 622
AT1G60130.1 | Symbols: no symbol available | no full name available | chr1:22174179-22176321 FORWARD LENGTH 600
AT1G57570.1 | Symbols: no symbol available | no full name available | chr1:21321717-21324284 FORWARD LENGTH 614
AT1G61230.1 | Symbols: no symbol available | no full name available | chr1:22578951-22580781 FORWARD LENGTH 505
AT5G35940.1 | Symbols: no symbol available | no full name available | chr5:14090825-14092808 FORWARD LENGTH 444
AT5G35950.1 | Symbols: no symbol available | no full name available | chr5:14104943-14106764 FORWARD LENGTH 444
AT1G52100.1 | Symbols: no symbol available | no full name available | chr1:19383291-19385123 REVERSE LENGTH 437
AT2G25980.1 | Symbols: no symbol available | no full name available | chr2:11077718-11079642 REVERSE LENGTH 449
AT1G33790.2 | Symbols: no symbol available | no full name available | chr1:12256990-12260432 FORWARD LENGTH 745
AT5G28520.1 | Symbols: no symbol available | no full name available | chr5:10493110-10494852 FORWARD LENGTH 453
AT1G52050.1 | Symbols: no symbol available | no full name available | chr1:19355106-19356748 REVERSE LENGTH 313
AT1G52060.1 | Symbols: no symbol available | no full name available | chr1:19359039-19360651 REVERSE LENGTH 314
AT1G52070.1 | Symbols: no symbol available | no full name available | chr1:19365140-19366900 REVERSE LENGTH 315
AT1G58160.1 | Symbols: JAX1 | JACALIN-TYPE LECTIN REQUIRED FOR POTEXVIRUS RESISTANCE 1 | chr1:21533724-21534440 FORWARD LENGTH 131
AT1G05760.2 | Symbols: RTM1 | restricted tev movement 1 | chr1:1723797-1724512 REVERSE LENGTH 198
AT1G05770.1 | Symbols: no symbol available | no full name available | chr1:1725547-1726250 REVERSE LENGTH 184
AT2G43740.1 | Symbols: no symbol available | no full name available | chr2:18126828-18127884 REVERSE LENGTH 309
AT1G52000.1 | Symbols: no symbol available | no full name available | chr1:19333352-19335700 REVERSE LENGTH 730
AT1G52030.1 | Symbols: F-ATMBP MBP1.2 MBP2 | myrosinase-binding protein 2 | chr1:19346090-19348282 REVERSE LENGTH 642
AT1G52040.1 | Symbols: MBP1 ATMBP | myrosinase-binding protein 1 | chr1:19350595-19352578 REVERSE LENGTH 462
AT3G16400.1 | Symbols: NSP1 ATNSP1 ATMLP-470 | nitrile specifier protein 1 NITRILE SPECIFIER PROTEIN 1 MYROSINASE-BINDING PROTEIN-LIKE PROTEIN-470 | chr3:5566516-5568330 FORWARD LENGTH 470
AT3G16410.1 | Symbols: NSP4 | nitrile specifier protein 4 | chr3:5572145-5574359 FORWARD LENGTH 619
AT3G16390.1 | Symbols: NSP3 | nitrile specifier protein 3 | chr3:5562602-5564356 FORWARD LENGTH 467
AT2G33070.3 | Symbols: ATNSP2 NSP2 | NITRILE-SPECIFIER PROTEIN 2 nitrile specifier protein 2 | chr2:14029350-14030934 REVERSE LENGTH 473
AT3G16470.1 | Symbols: JAL35 AtJAC1 JR1 | jacalin-related lectin 35 JACALIN-LECTIN LIKE 1 JASMONATE RESPONSIVE 1 | chr3:5596096-5597709 REVERSE LENGTH 451
AT5G46000.1 | Symbols: no symbol available | no full name available | chr5:18654319-18656376 REVERSE LENGTH 396
AT2G39310.1 | Symbols: JAL22 | jacalin-related lectin 22 | chr2:16414262-16416323 REVERSE LENGTH 458
AT2G39330.2 | Symbols: JAL23 | jacalin-related lectin 23 | chr2:16419787-16421717 REVERSE LENGTH 507
AT3G21380.1 | Symbols: no symbol available | no full name available | chr3:7528478-7530457 FORWARD LENGTH 460
AT3G16460.1 | Symbols: JAL34 | jacalin-related lectin 34 | chr3:5593029-5595522 FORWARD LENGTH 705
AT3G16420.1 | Symbols: PBPI JAL30 PBP1 | PYK10-binding protein 1 JACALIN-RELATED LECTIN 30 | chr3:5579560-5580674 FORWARD LENGTH 298
AT3G16430.1 | Symbols: JAL31 PBPII | jacalin-related lectin 31 | chr3:5581830-5582959 FORWARD LENGTH 296
AT3G16440.2 | Symbols: ATMLP-300B MLP-300B MEE36 | myrosinase-binding protein-like protein-300B maternal effect embryo arrest 36 | chr3:5585942-5587541 FORWARD LENGTH 323
AT3G16450.1 | Symbols: JAL33 | Jacalin-related lectin 33 | chr3:5588593-5589792 FORWARD LENGTH 300
AT1G19715.3 | Symbols: no symbol available | no full name available | chr1:6816934-6819432 REVERSE LENGTH 601
AT1G73040.1 | Symbols: no symbol available | no full name available | chr1:27475159-27475777 REVERSE LENGTH 176
AT3G59590.1 | Symbols: no symbol available | no full name available | chr3:22014167-22015768 FORWARD LENGTH 454
AT3G59610.1 | Symbols: no symbol available | no full name available | chr3:22019553-22021199 FORWARD LENGTH 521
AT3G59620.1 | Symbols: no symbol available | no full name available | chr3:22024039-22024496 FORWARD LENGTH 120
OsJAC1 Os12t0247700-01
HvJAC1 HORVU.MOREX.r2.5HG0398610.1.mrna1
TaJA1 A TraesCS2A02G033900.1
TaJA1 D TraesCS3D02G274000.1
TaJA1 B TraesCS2B02G100800.1
AT5G42655.2 | Symbols: no symbol available | no full name available | chr5:17102213-17102785 REVERSE LENGTH 190
AT1G07730.2 | Symbols: no symbol available | no full name available | chr1:2397527-2398696 REVERSE LENGTH 389
AT2G28670.1 | Symbols: ESB1 | ENHANCED SUBERIN 1 | chr2:12300380-12301723 REVERSE LENGTH 436
AT2G39430.1 | Symbols: no symbol available | no full name available | chr2:16463422-16464390 FORWARD LENGTH 322
AT3G55230.1 | Symbols: no symbol available | no full name available | chr3:20471824-20472744 FORWARD LENGTH 306
AT3G24020.1 | Symbols: no symbol available | no full name available | chr3:8678827-8679558 FORWARD LENGTH 243
AT4G13580.1 | Symbols: no symbol available | no full name available | chr4:7900427-7901161 FORWARD LENGTH 244
AT4G11190.1 | Symbols: no symbol available | no full name available | chr4:6826682-6827236 FORWARD LENGTH 184
AT4G11210.1 | Symbols: no symbol available | no full name available | chr4:6832691-6833245 FORWARD LENGTH 184
AT4G11180.1 | Symbols: DIR12 DP1 | dirigent protein 12 dirigent protein 1 | chr4:6820049-6820606 FORWARD LENGTH 185
AT1G64160.1 | Symbols: DIR5 AtDIR5 | dirigent protein 5 | chr1:23814063-23814611 FORWARD LENGTH 182
AT4G23690.1 | Symbols: AtDIR6 DIR6 | Arabidopsis thaliana dirigent protein 6 dirigent protein 6 | chr4:12339152-12339715 REVERSE LENGTH 187
AT5G42500.1 | Symbols: no symbol available | no full name available | chr5:16994359-16994916 REVERSE LENGTH 185
AT5G42510.1 | Symbols: no symbol available | no full name available | chr5:16998268-16998816 REVERSE LENGTH 182
AT1G22900.1 | Symbols: no symbol available | no full name available | chr1:8103910-8104491 REVERSE LENGTH 193
AT1G65870.1 | Symbols: no symbol available | no full name available | chr1:24503624-24504193 FORWARD LENGTH 189
AT3G58090.1 | Symbols: no symbol available | no full name available | chr3:21512479-21513905 REVERSE LENGTH 271
AT2G21110.1 | Symbols: no symbol available | no full name available | chr2:9050290-9050850 REVERSE LENGTH 186
AT4G38700.1 | Symbols: no symbol available | no full name available | chr4:18076583-18077155 REVERSE LENGTH 190
AT2G21100.1 | Symbols: no symbol available | no full name available | chr2:9048687-9049336 REVERSE LENGTH 187
AT1G58170.1 | Symbols: no symbol available | no full name available | chr1:21536188-21536745 FORWARD LENGTH 185
AT5G49030.3 | Symbols: OVA2 | ovule abortion 2 | chr5:19875091-19883251 REVERSE LENGTH 1279
AT5G49040.1 | Symbols: no symbol available | no full name available | chr5:19882676-19883251 REVERSE LENGTH 191
AT3G13662.1 | Symbols: no symbol available | no full name available | chr3:4467090-4468470 FORWARD LENGTH 173
AT3G13660.1 | Symbols: no symbol available | no full name available | chr3:4465135-4465512 FORWARD LENGTH 125
AT1G55210.1 | Symbols: no symbol available | no full name available | chr1:20598057-20598620 REVERSE LENGTH 187
AT3G13650.1 | Symbols: no symbol available | no full name available | chr3:4463056-4463616 FORWARD LENGTH 186
0.5

Figure S4: Molecular phylogenetic analysis of DIR and JRL proteins of *Arabidopsis thaliana* and MonocotJRLs by Maximum Likelihood method

The evolutionary history was inferred by using the Maximum Likelihood method based on the JTT matrix-based model [Jones et al 1992]. The tree with the highest log likelihood (-51885.82) is shown. The percentage of trees in which the associated taxa clustered together is shown next to the branches. Initial tree(s) for the heuristic search were obtained automatically by applying Neighbor-Join and BioNJ algorithms to a matrix of pairwise distances estimated using a JTT model, and then selecting the topology with superior log likelihood value. The tree is drawn to scale, with branch lengths measured in the number of substitutions per site. The analysis involved 80 amino acid sequences. There were a total of 2628 positions in the final dataset. Evolutionary analyses were conducted in MEGA7 [Kumar et al 2016]. JRLs are marked with green circles and DIR with orange circles. *Os*JAC1 and barley and wheat orthologs are located on a separate branch in the middle of the figure.

David T. Jones, William R. Taylor, Janet M. Thornton, The rapid generation of mutation data matrices from protein sequences, Bioinformatics, Volume 8, Issue 3, June 1992, Pages 275–282,
Sudhir Kumar, Glen Stecher, Koichiro Tamura, MEGA7: Molecular Evolutionary Genetics Analysis Version 7.0 for Bigger Datasets, Molecular Biology and Evolution, Volume 33, Issue 7, July 2016, Pages 1870–1874,

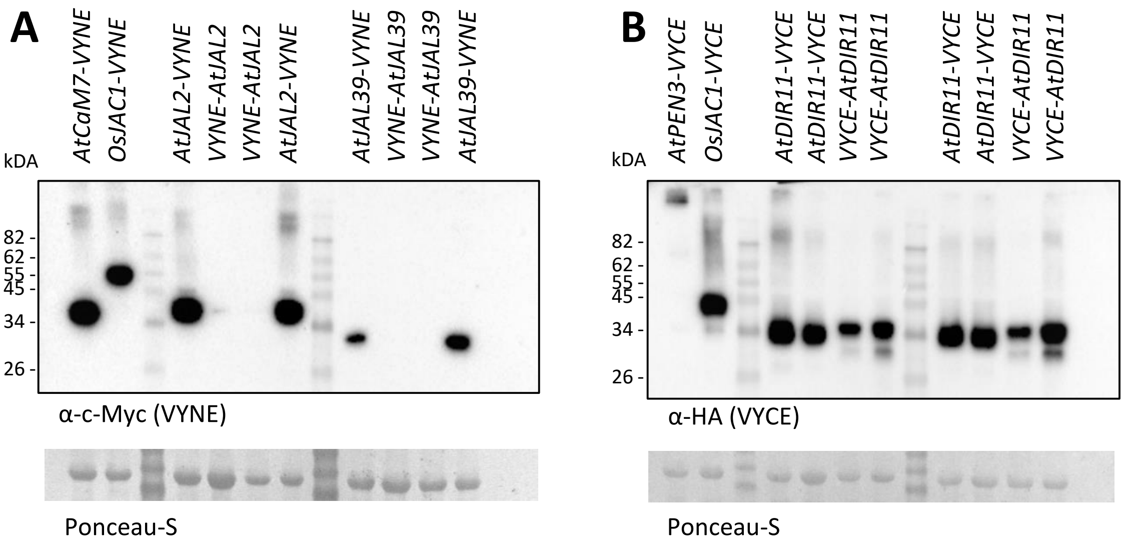

Figure S5: Protein abundance in the BiFC experiment with *At*DIR11 and *At*JAL2/*At*JAL39

A Western blot analyses was performed to check the abundance of co-expressed split-YFP fusion proteins in leaf extracts of *N. benthamiana*. Sampling was done from leaves used in the BiFC experiment shown in Fig. 5 and labelling of lanes is accordingly. The N-terminal part of YFP (VYNE) was detected with an anti-Myc antibody (α-c-Myc) A) whereas the C-terminal part of YFP (VYCE) was detected with an anti-HA antibody (α-HA) B). Membranes were stained with Ponceau-S after determination of chemiluminescence to visualize the total number of proteins in all lanes. The PiNK Prestained Protein Marker (NIPPON Genetics, Europe) was used to estmated the molecular mass of proteins as indicated.

Table S1: Primer used in the study.

For cloning from gDNA/cDNA			
**Gene**		**Primer name**	**Sequence 5'-3'**
OsJAC1	OsJAC1 (check transgenic lines)	OsJAC1_for	atctcgagatggctgatcccagcaag
Os12g0247700		OsJAC1_rev	atctcgagaagttagatcggctgcacgta
	Dirigent-Domain	OsDIR_rev	atctcgagttattcatgagatgaaccccatgg
	JRL-Domain	OsJAC_for	tactagtatgttaaaagggtcacagtgccct
TaJA1	Dirigent-Domain	TaJA1_DIR_for	tagcggccgcatggccaatttccagataactc
AY372111.1		TaJA1_DIR_rev	atctcgagttatccatttccaccccatg
	JRL-Domain	TaJA1_JAC_for	tactagtatgtgtcccatgctgaaaggtt
		TaJA1_JAC_rev	atggatccttagagagggtgcacgtagaca
HvJAC1	Dirigent-Domain	HvAK86_DIR_for	tagcggccgcatggccaactcctccaact
AK369786		HvAK86_DIR_rev	atctcgagttaccttgatgctttaatcatgttttg
	JRL-Domain	HvAK86_JAC_for	tactagtatgtgccccttgctgaaagg
		HvAK86_JAC_rev	atggatccttaaacaggctgcaagtatatacca
AtJAX1		AtJAX1_for	taggatccatggctacaccatcaggt
At1G58160		AtJAX1_rev	atctcgagttacttagcaggagaaagc
ATJAX1-INTRON		AtJAX1_vI_r	tatggttaagatcaaaccactcggtgaaattgtga
		AtJAX1_nI_f	caatttcaccgagtggtttgatcttaaccatacat
ATJAX1 BAY-0		AtJAX1-Bay_allelsp	atggagtccatgaaggagtgttac
ATJAX1 COL-0		AtJAX1-Col_allelsp	atggagtccatgaaggagtgtgat
ATJAX1 BAY-0/COL-0		AtJAX1-rev	ttacttagcaggagaaagcggtg
ATDIR19		AtDir_for	taggatccgaaagtcatctctctccagtctcca
At1G58170		AtDir_rev	atctcgagttagtagtgcaaaagataacaatt
ATDIR19-ATJAX1-FUSION		DIR1_link_full_r	ttcatgagatgaaccccatggcccaatcttggtgacagggcactgtgacccttttaagagaggaca

		JAX1_link_full_f	ccctgtcaccaagattgggccatggggttcatctcatgaagctacaccatcaggttcaaat
ATRTM1		At1g05760_for	atgaagataggacctgtagggaa
At1g05760		At1g05760_rev	tcagcccagtacaatttttgact
ATJAL2		At1g05770_for	cacataaagaagaaaatggaagggaaaatc
At1g05770		At1g05770_rev	ttaatacacaatttcatttaacccaaaaca
ATJAL3		At1g19715_for	aatctgaggatggtattaacatcatcttat
At1g19715		At1g19715_rev	cagaagagcttgaaacaactagc
ATJAL19		At1g73040_for	atggatcaacaacaacaaggtga
At1g73040		At1g73040_rev	tcatgctactatccttgtgagcc
ATJAL24		At2g43730_for	ttcaatgaaatctcgtaatagggagat
At2g43730		At2g43730_rev	ttaatccaagaaaacttttatcaggcacaa
ATJAL25		At2g43740_for	ctgctatattctaaatgaaatcccgtgg
At2g43740		At2g43740_rev	gatctaatccaagagatttctgaaaagacg
ATJAL39		At3g59620_for	atggcatttcgtatgagccga
At3g59620		At3g559620_rev	tcaattaaaaccaccgaagtcaaagg
	(to exclude intron)	AtJal39 fw1	ATGGCATTTCGTATGAGCC
	(to exclude intron)	AtJal39 rv1	CTCTGCTCGAGCACCC
	(to exclude intron)	AtJal39_fw2	GGGTGCTCGAGCAGAG TTTTGGCTGAGAGACGATG
	(to exclude intron)	AtJal39_rv2	TCAATTAAAACCACCGAAGTC
ATDIR11	At1g22900	At1g22900_for	atgttacaaataacgaacatgg
		At1g22900_rev	ttaatgccaaatgaagacatta

For cloning into TOX expression vector pIPKTA09			
**Gene**			**Sequence 5'-3'**
OsJAC1	Os12g0247700		Weidenbach et al. 2016
TaJA1	AY372111.1		Weidenbach et al. 2016
HvJAC1	AK369786		Weidenbach et al. 2016
ATRTM1	At1g05760	At1g05760_NotI_f	atgcggccgcatgaagataggacctgtagggaa
		At1g05760_XhoI_r	atctcgagtcagcccagtacaatttttgact
ATJAL2	At1g05770	At1g05770_NotI_f	atgcggccgccacataaagaagaaaatggaagggaaaatc
		At1g05770_BamHI_r	atggatccttaatacacaatttcatttaacccaaaaca
ATJAL3	At1g19715	At1g19715_NotI_f	atgcggccgcaatctgaggatggtattaacatcatcttat
		At1g19715_BamHI_r	atggatcccagaagagcttgaaacaactagc
ATJAL19	At1g73040	At1g73040_NotI_f	atgcggccgcatggatcaacaacaacaaggtga
		At1g73040_XhoI_r	atctcgagtcatgctactatccttgtgagcc
ATJAL24	At2g43730	At2g43730_NotI_f	atgcggccgcttcaatgaaatctcgtaatagggagat
		At2g43730_Xho_rI	atctcgagttaatccaagaaaacttttatcaggcacaa
ATJAL25	At2g43740	At2g43740_NotI_f	atgcggccgcctgctatattctaaatgaaatcccgtgg
		At2g43740_XhoI_r	atctcgaggatctaatccaagagatttctgaaaagacg
ATJAL39	At3g59620	At3g59620_NotI_for	atgcggccgcatggcatttcgtatgagccga
		At3g59620_BamHI_rev	atggatcctcaattaaaaccaccgaagtcaaagg
ATDIR11	At1g22900	AT1G22900_BamHI_f	ttggatccatgttacaaataacgaacatgg
		AT1g22900_XhoI_r	ttctcgagttaatgccaaatgaagacatta

For Gateway cloning (pDONR207 and BiFC expression vectors (GW-VYCE, GW-VYNE, VYCE-GW, VYNE-GW))			
**Gene**			**Sequence 5'-3'**
OsJAC1	OsJAC1attBfw		gggg aca agt ttg tac aaa aaa gca ggc ttc **ATGGCTGATCCCAGCAAGCTG**
	OsJAC1attB_c-st_rv	-Stop	gggg ac cac ttt gta caa gaa agc tgg gtt **GATCGGCTGCACGTAGACACC**
Dirigent-Domain-stop	OsDir_C-Fluoro_for		ggggacaagtttgtacaaaaaagcaggcttcgaaggagatagaaccatgctatggctgatcccagcaag
	OsDir_C-Fluoro_r		ggggaccactttgtacaagaaagctgggtgatgagatgaaccccatggcc
JRL-Domain+stop	OsJac_N-Fluoro_f		ggggacaagtttgtacaaaaaagcaggcttcttaaaagggtcacagtgccct
	OsJac_N-Fluoro_r		gggaccactttgtacaagaaagctgggttttagttagatcggctgcacgtaga
JRL-Domain-stop	OsJac_C-Fluoro_f		ggggacaagtttgtacaaaaaagcaggcttcgaaggagatagaaccatgttaaaagggtcacagtgccct
	OsJac_C-Fluoro_r		ggggaccactttgtacaagaaagctgggtggatcggctgcacgtagacacc
AtDIR19	AtDIR19_C-Fluoro_f		ggggacaagtttgtacaaaaaagcaggcttcgaaggagatagaaccatgggcagttttctctcc
	AtDIR19_C-Fluoro_r	-Stop	ggggaccactttgtacaagaaagctgggtggtagtgcaaaagataacaatt
ATJAX1	AtJAX1_C-Fluoro_f	-Stop	ggggacaagtttgtacaaaaaagcaggcttcgaaggagatagaaccatggctacaccatcaggt
	AtJAX1_C-Fluoro_r		ggggaccactttgtacaagaaagctgggtgcttagcaggagaaagcggt
	AtJAX1_N-Fluoro_f	+Stop	ggggacaagtttgtacaaaaaagcaggcttcatggctaaccatcaggt
	AtJAX1_N-Fluoro_r		ggggaccactttgtacaagaaagctgggttttacttagcaggagaaagc
AtJAL2	At1g05770_att_f		GGGGACAAGTTTGTACAAAAAAGCAGGCTTCcacataaagaagaaaatggaagggaaaatc
	At1g05770_c-t_att_r	-Stop	GGGGACCACTTTGTACAAGAAAGCTGGGTGttaatacacaatttcatttaacccaaaaca
	At1g05770_att_r		GGGGACCACTTTGTACAAGAAAGCTGGGTTttaatacacaatttcatttaacccaaaaca
AtJAL39	AtJal39_att_fw		GGGG ACA AGT TTG TAC AAA AAA GCA GGC TTC ATGGCATTTCGTATGAGCC
	AtJal39_att_rv		GGGG AC CAC TTT GTA CAA GAA AGC TGG GTT TCAATTAAAACCACCGAAGTC

	jal39 att_c-stp_rv	-Stop	GGGG AC CAC TTT GTA CAA GAA AGC TGG GTT ATTAAAACCACCGAAGTCAAAG
	At1g22900_att_f		ggggacaagtttgtacaaaaaagcaggcttcatgttacaaataacgaacatgg
	At1g22900_c-t_att_r	-Stop	ggggaccactttgtacaagaaagctgggtgatgccaaatgaagacatta
	At1g22900_att_r		GGGGACCACTTTGTACAAGAAAGCTGGGTTttaatgccaaatgaagacatta

**qPCR primer**			
	**Primer name**	**Sequence 5'-3'**	
OsJAC1 (Oryza sativa)	OsJAC1_qPCR_for	atccatgggttttgtcctctc	
	OsJAC1_qPCR_rev	agacgctttggagactcagtg	
AtActin (Arabidopsis thaliana)	AtActin_for	gctaacattgtgctcagtggtgg	
	AtActin_rev	ggtgcaacgaccttaatcttcat	
